# The association between pain and diagnosis-specific long-term sickness absence at different levels of body mass index – a register-linked cohort study

**DOI:** 10.1186/s12889-026-26696-8

**Published:** 2026-02-17

**Authors:** Pi Fagerlund, Jatta Valkonen, Mari-Liis Kalima, Teemu Miettinen, Anna C. Svärd, Tea Lallukka

**Affiliations:** 1 Department of Public Health, Faculty of Medicine, University of Helsinki, Helsinki, Finland; 2 Department of Anaesthesiology, Intensive Care and Pain Medicine, University of Helsinki, Helsinki University Hospital, Helsinki, Finland

**Keywords:** Chronic pain, Absenteeism, Work, Obesity

## Abstract

**Background:**

Chronic (≥ 3 months) pain and obesity (body mass index [BMI] ≥ 30 kg/m^2^) are both associated with long-term sickness absence (LTSA). This study aimed to examine the contribution of acute (< 3 months) and chronic pain to total and diagnosis-specific LTSA (> 10 working days) at different levels of BMI among young and early midlife employees.

**Methods:**

Helsinki Health Study questionnaire data covering 19–39-year-old employees were collected in 2017 (*n* = 4091). Pain was classified as no pain, acute pain, or chronic pain. BMI was classified as healthy weight (BMI 18.5–24.9 kg/m^2^), overweight (BMI 25.0–29.9 kg/m^2^), or obesity. Diagnosis-specific follow-up data on LTSA covering 5 years after the survey collection were obtained from the Social Insurance Institution of Finland’s registers. Negative binomial regression analyses were conducted, adjusting for key covariates.

**Results:**

Acute (RR 1.57, 95% CI 1.19 − 2.08) and chronic (RR 2.05, 95% CI 1.52 − 2.75) pain, and overweight (RR 1.42, 95% CI 1.09 − 1.85) and obesity (RR 2.20, 95% CI 1.58 − 3.05) were all associated with LTSA after adjusting for age and gender. The strongest association with LTSA was found for coexistent chronic pain and obesity (RR 3.49, 95% CI 1.89 − 6.43). Adjusting further for other sociodemographic, lifestyle and work-related factors marginally attenuated the associations. Having coexistent acute pain and a healthy weight or having no pain and overweight were not associated with LTSA.

**Conclusions:**

Employees with coexistent pain and overweight/obesity have an increased risk of LTSA, particularly employees with coexistent chronic pain and obesity. These employees could benefit from targeted primary and secondary preventive measures at the workplaces and in occupational healthcare to reduce LTSA.

**Supplementary Information:**

The online version contains supplementary material available at 10.1186/s12889-026-26696-8.

## Background

Chronic pain, or pain that persists or recurs for three months or longer, is a central symptom in musculoskeletal disorders and a common comorbidity to mental disorders such as anxiety and depression [1–3]. Chronic pain contributes to disability globally, and 20% of Finnish municipal employees under 40 years are estimated to have chronic pain, which is also associated with long-term sickness absence (LTSA) in this age group [4, 5]. Musculoskeletal disorders among young people are expected to increase on a global level over the coming decades, with increasing obesity being a central contributing factor [6]. 

Obesity affects close to 30% of Finnish working-age adults, and approximately two thirds of men and half of women have at least overweight [7]. Overweight and obesity are less common in younger age groups compared to older ones, but they are increasing among them as well [7]. Obesity increases the risk of comorbidities that may impact work ability, and previous research shows that obesity is associated with poorer labour market outcomes, such as lower income, LTSA and disability retirement [8–11]. Among Finnish midlife and ageing employees, obesity has been associated with LTSA, particularly due to musculoskeletal disorders but also due to mental disorders [12].

LTSA reflects employees’ ill-health and reduced work ability, contributes to costs and productivity loss, and is associated with an increased risk of later disability retirement [13, 14]. As long-term work disability has the most far-reaching consequences for young and early midlife employees with a long work-life ahead of them, focusing on preventing LTSA in this group is particularly important. Mental disorders, followed by musculoskeletal disorders, contribute most to LTSA among young employees in Finland [15].

Coexistent chronic pain and obesity are common, and the relationship is bidirectional so that either condition risks to worsen or predispose to the other [16]. The prevalence of chronic pain increases with higher BMI, and factors related to biomechanics, inflammation, sleep, mood, and lifestyle are suggested to contribute to the link between these conditions [16]. Despite the common coexistence, studies lack on how pain is associated with LTSA at different BMI levels. Understanding this connection is important for enabling identification of employees at highest risk of SA and who could benefit from support and targeted interventions in occupational healthcare and at workplaces to prevent LTSA, and to support work ability and labour market attachment.

This study aimed to examine how pain is associated with subsequent LTSA at different BMI levels among initially 19–39-year-old Finnish employees, accounting for sociodemographic and lifestyle factors and workload. LTSA data were collected over 5 years and analysed as total and ICD-10 diagnosis-specific LTSA days. 

## Methods

### Data collection and study population

**Fig. 1:**
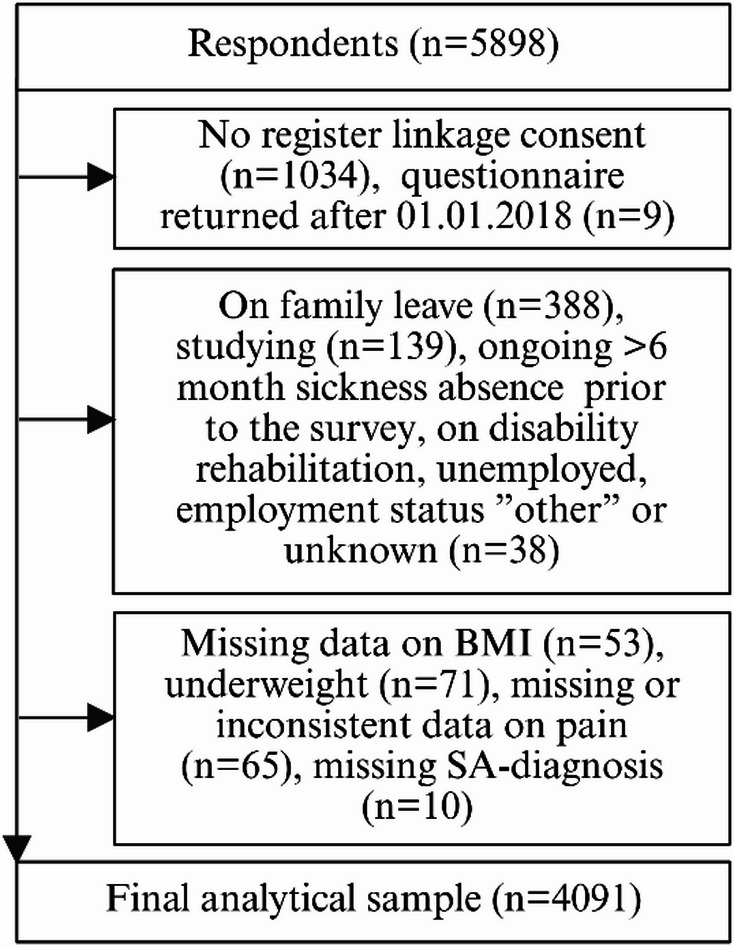
Flowchart diagram of the selection of the study population. BMI body mass index, SA sickness absence. Created with *BioRender.com*

The data consisted of cross-sectional questionnaire data collected in fall 2017 as part of the Helsinki Health Study, combined with longitudinal register data from the Social Insurance Institution of Finland. The Helsinki Health study is a cohort study that follows the health and working conditions of employees of the City of Helsinki, Finland. The questionnaire was not developed for this study only. The target population consisted of 11,459 19–39-year-old employees of the City of Helsinki, Finland, who had had an at least 50% work contract for over 4 months prior to the survey. Answers were obtained by online questionnaires sent to employees’ work-email (58%), by mail (29%), and by shorter phone interviews among non-respondents of the online survey and mailed survey (13%) [17]. The overall response rate was 51.5%, and the data were found to be broadly representative of the target population [17]. Only respondents who had given their informed consent to register linkage (82%) were included in this study (Fig. 1). For obtaining the final study population, a set of exclusion criteria was applied. Nine employees who had returned the questionnaire after 1st of January 2018 were excluded, as a 5-year LTSA follow-up was not available for them. Employees who were not working full-time or part-time when the survey was conducted were excluded. Respondents with missing data on height, weight or pain or inconsistent data on pain were excluded. Respondents who were underweight (BMI < 18.5 kg/m^2^) were excluded as this group was small (1.5%, *n* = 71), and they had more SA than those with healthy weight (BMI 18.5–24.9 kg/m^2^). Respondents who had LTSA but lacked diagnosis data (*n* = 10) were also excluded. The final study population consisted of 4,091 employees.

### Variables

#### Long-term sickness absence

LTSA data were obtained from the Social Insurance Institution of Finland’s national register which includes sickness absence spells that exceed the 10 initial workdays of a sickness absence spell for up to 300 workdays for the same diagnosis within a 2-year period, and thus, entitle to sickness allowance [18]. LTSA that entitles to sickness allowance from the Social Insurance Institution requires an ICD-10 diagnosis set by a physician. The data are collected irrespective of the employer at the time of the LTSA. The outcome measure LTSA was computed as days of sickness absence exceeding 10 initial workdays and was collected over 5 years starting from the day after the questionnaire was returned. To facilitate comparison, the obtained number of LTSA days were converted into LTSA days per 10 person years and further categorized by the ICD-10 diagnosis entitling to LTSA: psychiatric (F00–F99), musculoskeletal (M00–M99), and other diagnoses.

#### Pain and body mass index

Pain was enquired by asking “Are you suffering from any pains or aches right now?” Those who responded ‘yes’ were further asked “When did the pain begin?” and were given the answer options “up to 3 months ago” and “more than 3 months ago”. Based on these questions, pain status was classified as ’no pain’, “acute pain’, and ’chronic pain’, following previous procedures [1]. Current body height and weight were self-reported by the respondents in the questionnaire. BMI (weight in kilograms/ height squared in meters) was computed based on the responses and classified as ’healthy weight’ 18.5–24.9 kg/m^2^, ’overweight’ 25.0–29.9 kg/m^2^, and ’obesity’ ≥30 kg/m^2^. Pain and BMI levels were further combined to obtain nine categories: ’no pain and healthy weight’, ’acute pain and healthy weight’, ’chronic pain and healthy weight’, ’no pain and overweight’, ’acute pain and overweight’, ’chronic pain and overweight’, ’no pain and obesity’, ’acute pain and obesity’, and ’chronic pain and obesity’.

#### Covariates

Sociodemographic, lifestyle and work-related factors were included as covariates based on their previously shown associations with sickness absence, pain and BMI [19–23]. Covariates were selected based on the data available also from the shortened phone interviews, to have more representative data [24–26]. Age was classified as < 30 or ≥ 30 years for descriptive purposes but analyzed as a continuous variable in the regression models. Gender was reported and classified as ’woman’ (77% of the study population, [Table 1]) or ’man’. Marital status was dichotomized as ’married or cohabiting’ or “single” (unmarried, widowed and divorced). Education level was classified as ’low’ (upper secondary school or lower), “intermediate” (bachelor’s degree or equivalent) or ’high’ (master’s degree or higher). Smoking was categorized as ’never or stopped’ or ’daily or occasionally’. The frequency of alcohol use was enquired on a ten-level scale and classified as ‘> weekly’ or ‘≤weekly’. Average sleep duration was classified as’<7 h’, ’7–8 h’, and’>8 h’. Heavy physical and mental workload are associated with higher rates of workdays lost to work disability, why physical and mental workload were considered as covariates and enquired by the question “What is your job like”, and response options on a four-level scale ranged from ’very light’ to ’very strenuous’ [23, 27]. For physical workload, a dichotomized variable was created where ’very light’ to ’quite light’ were classified as ’light’, and ’quite strenuous’ to ’very strenuous’ were classified as ’heavy’. For mental workload, the cut-off between ’light’ and ’heavy’ was set between ’quite strenuous’ and ’very strenuous’ due to a high proportion of employees reporting strenuous workload. Fresh vegetable consumptions served as proxy measure for healthy diet and was enquired from a 14-item food frequency questionnaire and classified as’≥ daily’ and’< daily’.

**Table 1:** Baseline characteristics of the Helsinki health study population (*N* = 4091) in percentages and by long-term sickness absence (LTSA) days per 10 person years over a 5-year follow-up 2017–2022

	*n* (%)	Mean LTSA days per 10 person years
Gender		
Woman	3138 (77)	39
Man	953 (23)	34
Age		
< 30 years	1311 (32)	43
≥ 30 years	2780 (68)	35
Education level		
High	1219 (30)	22
Intermediate	1876 (46)	42
Low	996 (24)	49
Smoking		
Never or stopped	3099 (76)	34
Daily or occasionally	992 (24)	48
Alcohol use		
≤ Weekly	2817 (69)	41
> Weekly	1,134 (28)	29
Missing	140 (3)	37
Sleep duration		
< 7 h	821 (20)	46
7–8 h	2901 (71)	34
> 8 h	369 (9)	47
Vegetable consumption		
≥ Daily	2905 (71)	42
< Daily	1,186 (29)	36
Physical workload		
Low	2701 (66)	34
High	1,390 (34)	45
Mental workload		
Low	3428 (84)	35
High	663 (16)	53

Missing observations were in general few (0.9% or less), except for alcohol use (*n* = 140, 3.4%). Missing observations were merged with the reference group, except for education level and alcohol use. Missing education level was merged with the low education category as non-respondents tend to have a lower education level, as in previous studies [28]. Due to many missing variables in alcohol use, respondents with missing responses were classified as their own category (*n* = 143). These imputations were done to avoid selection in the analytical sample.

### Statistical methods

Variables covering baseline characteristics of the study population were cross tabulated for descriptive purposes and presented as percentages and mean LTSA days per 10 person years. Of the study population, 67% had no LTSA during the 5-year follow-up. Due to the zero-inflation of the outcome measure, negative binomial regression analyses were conducted to examine the association of pain with LTSA at different levels of BMI. Respondents who reported no pain and had a healthy weight were defined as the reference group. Results were reported as rate ratios (RR) with their 95% confidence intervals (CI). Four regression models were fitted. Model 1 was adjusted for age and gender. Model 2 was adjusted for age, gender, marital status and education level and Model 3 further for smoking, alcohol use, vegetable consumption and sleep duration. Model 4 was adjusted for age, gender, marital status, education level, physical workload and mental workload. A full mutually adjusted model was not included to avoid over adjustment.

Gender differences in the prevalence of pain, overweight, obesity and SA rates are known and thus, analyses of gender interaction to the association of pain with LTSA at different levels of BMI were performed [4, 12]. A gender interaction to the association with LTSA was found for the classes ‘no pain and obesity’ (*p* = 0.01), and ‘acute pain and overweight’ (*p* = 0.013). Apart from these differences, associations were similar for the two genders (Supplementary Table 1). The overall gender differences were considered minor and the number of men was relatively small, why both genders were analyzed together, and the regression analyses were adjusted for gender.

The association between pain and diagnosis-specific LTSA at different levels of BMI were analyzed to clarify potential differences in reasons for LTSA in subgroups with different pain and BMI status (Supplementary Table 2). Associations of pain at different levels of BMI by employment sector were analyzed to clarify potential employment sector differences (Supplementary Table 3).

### Ethical considerations

The study protocol of the Helsinki Health Study has obtained a positive statement from the ethics committee of the Faculty of Medicine, University of Helsinki, and has been approved by the City of Helsinki. The respondents have, by participating in the Helsinki Health Study, agreed to the questionnaire data being used for scientific research purposes. The data are handled in line with the data protection statement of the Helsinki Health Study [29]. As part of the Helsinki Health Study, this observational study did not require a separate ethical approval, however, ethical aspects of the study have been considered in line with the Declaration of Helsinki 1964.

## Results

### Descriptive results

Of all participants, 43% reported acute pain and 19% reported chronic pain at inclusion (Table 2). Correspondingly, 59% had a healthy weight, whereas 27% had overweight and 15% obesity. Respondents with chronic pain had twice as many LTSA days (57 vs. 28 days per 10 person years) during the 5-year follow-up compared to those who had no pain. Similarly, employees with obesity had twice as many LTSA days as those with healthy weight (63 vs. 30 days per 10 person years).

**Table 2:** Prevalence of pain status and body mass index (BMI) levels separately and combined, in total and by gender. Combined pain status and BMI levels by long-term sickness absence (LTSA) days per 10 person years during a 5-year follow-up. *N* = 4091

	Total	Women	Men	LTSA days per 10 pers.yrs^a^			
	*n* (%)	*n* (%)	*n* (%)	All diagnoses	F-diagnoses	M-diagnoses	Other diagnoses
Pain status							
No pain	2388 (58.4)	1759 (56.1)	629 (66.0)	28	16	3	10
Acute pain	911 (22.3)	750 (23.9)	161 (16.9)	45	23	7	15
Chronic pain	792 (19.4)	629 (20.0)	163 (17.1)	57	28	9	19
BMI							
Healthy weight	2391 (58.5)	1952 (62.2)	439 (46.1)	30	16	4	10
Overweight	1100 (26.9)	724 (23.1)	376 (39.5)	40	19	6	15
Obesity	600 (14.7)	462 (14.7)	138 (14.5)	63	36	8	19
Pain status by BMI							
No pain, healthy weight	1469 (35.9)	1165 (37.1)	304 (31.9)	25	14	2	9
Acute pain, healthy weight	510 (12.5)	438 (14.0)	72 (7.6)	37	17	6	14
Chronic pain, healthy weight	412 (10.1)	349 (11.1)	63 (6.6)	40	21	6	13
No pain, overweight	617 (15.1)	371 (11.8)	246 (25.8)	25	10	3	12
Acute pain, overweight	253 (6.2)	190 (6.1)	63 (6.6)	50	29	7	15
Chronic pain, overweight	230 (5.6)	163 (5.2)	67 (7.0)	69	32	12	25
No pain, obesity	302 (7.4)	223 (7.1)	79 (8.3)	52	34	3	15
Acute pain, obesity	148 (3.6)	122 (3.9)	26 (2.7)	64	33	12	19
Chronic pain, obesity	150 (3.7)	117 (3.7)	33 (3.5)	84	42	15	26

*LTSA* long-term sickness absence, *Pers.yrs* Person years

^a^Total number of LTSA days by all diagnoses, psychiatric diagnoses (F-diagnoses), musculoskeletal diagnoses (M-diagnoses), and other diagnoses

When examining pain by LTSA days at different levels of BMI, individuals with no pain and healthy weight had the least LTSA days (25 days per 10 person years). Employees with coexistent acute pain and overweight/obesity had more LTSA days than employees with healthy weight and no pain. Participants with chronic pain and obesity had most LTSA days (84 days per 10 person years).

### Associations of pain and body mass index with long-term sickness absence

In the regression analyses (Table 3), both acute (RR 1.57, 95% CI 1.19 − 2.08) and chronic (RR 2.05, 95% CI 1.52 − 2.75) pain were associated with LTSA after adjusting for age and gender, when employees with no pain were the reference group. Similarly, overweight (RR 1.42, 95% CI 1.09 − 1.85) and obesity (RR 2.20, 95% CI 1.58 − 3.05) were associated with LTSA when healthy weight was the reference group. When examining combinations of pain and BMI statuses, most categories showed increased rates of LTSA compared to those with no pain and healthy weight. However, those with no pain and overweight did not have a significantly increased rate of LTSA (RR 1.02, 95% CI 0.73 − 1.44). The strongest association with LTSA was seen for employees with coexistent chronic pain and obesity (RR 3.49, 95% CI 1.89 − 6.43). Adjusting further for marital status, education level, lifestyle factors and workload only marginally affected the results.

**Table 3:** Associations of pain and body mass index (BMI) status with long-term sickness absence (all diagnoses), *N* = 4091

	Model 1	Model 2	Model 3	Model 4
	RR (95% CI)	RR (95% CI)	RR (95% CI)	RR (95% CI)
Pain status				
No pain	1	1	1	1
Acute pain	1.57 (1.19 − 2.08)	1.54 (1.16 − 2.03)	1.59 (1.20 − 2.11)	1.57 (1.17 − 2.11)
Chronic pain	2.05 (1.52 − 2.75)	2.00 (1.49 − 2.69)	2.11 (1.57 − 2.85)	1.96 (1.43 − 2.68)
BMI				
Healthy weight	1	1	1	1
Overweight	1.42 (1.09 − 1.85)	1.37 (1.05 − 1.79)	1.41 (1.08 − 1.84)	1.30 (0.96 − 1.75)
Obesity	2.20 (1.58 − 3.05)	1.92 (1.38 − 2.69)	1.94 (1.39 − 2.71)	1.78 (1.24 − 2.54)
Pain status by BMI				
No pain, healthy weight	1	1	1	1
Acute pain, healthy weight	1.41 (0.98 − 2.04)	1.41 (0.98 − 2.04)	1.45 (1.00 − 2.09)	1.48 (1.01 − 2.17)
Chronic pain, healthy weight	1.57 (1.05 − 2.34)	1.55 (1.04 − 2.31)	1.62 (1.08 − 2.42)	1.86 (1.24 − 2.80)
No pain, overweight	1.02 (0.73 − 1.44)	1.03 (0.74 − 1.45)	1.05 (0.75 − 1.48)	1.27 (0.85 − 1.91)
Acute pain, overweight	2.06 (1.27 − 3.35)	2.00 (1.22 − 3.25)	2.11 (1.30 − 3.45)	1.96 (1.15 − 3.33)
Chronic pain, overweight	3.03 (1.82 − 5.04)	2.86 (1.72 − 4.76)	3.08 (1.84 − 5.13)	2.19 (1.24 − 3.87)
No pain, obesity	2.17 (1.38 − 3.41)	1.85 (1.17 − 2.92)	1.84 (1.17 − 2.91)	1.53 (0.93 − 2.51)
Acute pain, obesity	2.60 (1.41 − 4.82)	2.31 (1.25 − 4.28)	2.41 (1.30 − 4.49)	2.59 (1.36 − 4.91)
Chronic pain, obesity	3.49 (1.89 − 6.43)	3.29 (1.77 − 6.11)	3.45 (1.86 − 6.43)	3.39 (1.74 − 6.59)

Model 1 age, gender

Model 2 age, gender, marital status, education level

Model 3 age, gender, marital status, education level, smoking, alcohol use, vegetable consumption, sleep duration

Model 4 age, gender, marital status, education level, physical workload, mental workload

*RR* Rate ratio, *CI* Confidence interval

## Discussion

### Main findings

We examined the association of acute and chronic pain with LTSA at different levels of BMI (healthy weight, overweight, or obesity) among young and early midlife Finnish employees. Employees with acute or chronic pain and overweight/obesity had more LTSA days than those who reported no pain and had a healthy weight. Highest rates of LTSA days were found for employees with coexistent chronic pain and obesity, who had more than three times as many LTSA days as employees with no pain and healthy weight. The associations of acute or chronic pain and overweight/obesity with LTSA were marginally explained by sociodemographic, lifestyle and work-related factors. Having overweight without pain was not associated with LTSA.

### Comparison to previous findings

LTSA is a significant indicator of work disability and is important to prevent due to its costliness and association with later disability retirement [13]. Pain is a core symptom in musculoskeletal disorders, although common also in non-musculoskeletal conditions [30]. A recent study exploring the global burden of musculoskeletal disorders among 15–39-year-old adults found an increase in musculoskeletal disorders between 1990 and 2019 and projected a further increase over the coming decades, with increasing prevalence of obesity being a central contributing factor [6]. The projected increase in disability adjusted life-years related to musculoskeletal disorders was estimated to affect women to a higher extent than men [6]. Additionally, a study based on the same cohort as the current study showed that chronic pain and pain involving multiple body sites were associated with both all-cause total sickness absence and with LTSA [4]. The projected higher burden of musculoskeletal disorders among women and the role of obesity are important to note in efforts to prevent pain-related LTSA and musculoskeletal disorders among public sector employees, who predominately are women.

That elevated BMI is a risk factor for sickness absence has been demonstrated in many previous studies [9, 12, 21, 31]. In a systematic review from 2009, Duijvenbode et al. showed that obesity, as compared to overweight, was a more consistent risk factor for sickness absence, particularly for LTSA [31]. The evidence for an association between overweight and sickness absence showed inconsistency but indicated a more consistent association for longer, over seven days long, sickness absence spells [31]. Similarly, a systematic review from 2009 by Neovius et al. showed that employees with obesity had more sickness absence, particularly LTSA, than their peers with healthy weight [9]. European employees with obesity had on average ten sickness absence days per person years more sickness absence than employees with a healthy weight [9]. A review study from 2024 also suggested that individuals with obesity have lower income on average and have an increased risk of disability retirement, but that there are differences in this association by gender, ethnicity and study setting [10]. A Finnish study conducted among midlife and ageing employees showed an association between weight gain, overweight and obesity with LTSA due to musculoskeletal disorders among women [12]. In the same study, obesity among women and weight gain among men with overweight were associated with LTSA due to psychiatric disorders [12].

Young employees have a long career ahead of them, and thus, supporting their work ability and labor market attachment is crucial considering the ageing of the population [32]. Findings from this study support earlier findings of pain and elevated BMI as risk factors for all-cause LTSA but also show that coexistent pain and overweight /obesity confer a particularly high risk of LTSA also among young and early midlife employees. Among under 40-year-old employees with pain, overweight and obesity have previously been associated with belonging to a high LTSA trajectory [25]. Similarly, in as study from 2014, Haukka et al. found overweight and obesity to be associated with sickness absence due to musculoskeletal pain among Finnish kitchen workers during a two-year follow-up [33]. The results thus suggest that in the early phase of career, pain and overweight/obesity—particularly if coexistently occurring—should be addressed as risk factors for declining work ability and motivate provision of support within occupational health care and at the workplace. However, due to the extent of chronic pain and overweight/obesity as public health issues, also broader preventive efforts to prevent pain and overweight/obesity in the younger workforce are needed. As psychiatric disorders are the most common reason for LTSA among Finnish employees, and the proportion of mental health-related LTSA is highest among younger employees, also efforts to prevent psychiatric comorbidities that may affect and exacerbate the prognosis and impact of pain and overweight/obesity may be needed [34].

This study considered diagnosis-specific LTSA and found that having no pain and obesity, or chronic pain and obesity were associated with LTSA due to psychiatric diagnoses (Supplementary Table 2). Considering the common comorbidity between depression, chronic pain, and obesity, mental health is likely to mediate part of the association of pain and obesity with LTSA, particularly in this age group [2, 35, 36]. Due to this, the regression analyses were not adjusted for mental health. Post-hoc analyses showed, however, that employees with chronic pain and obesity had a lower median score (70 points) on the RAND-36 emotional well-being scale (0–100) that measures symptoms of anxiety and affective disorders, as compared to the entire study population (76 points) and those with no pain and healthy weight (80 points). Anxiety, depression and sleep disorders are known to commonly coexist alongside chronic pain and overweight/obesity, and the conditions may exacerbate each other, complicate the treatment and contribute to further decreased work ability. This highlights the importance of early intervention to prevent chronification of pain and overweight/obesity, as well as importance of treatment that is holistic and considers comorbidities. Although more common among older adults, sickness absence due to musculoskeletal disorders are also common among young and early midlife Finnish employees [37]. In this study, chronic pain was associated with LTSA due to musculoskeletal disorders irrespective of BMI, when compared to employees with no pain and healthy weight. Acute pain was also associated with LTSA due to musculoskeletal disorders among employees with healthy weight, overweight and obesity (Supplementary Table 2). Pain is a central symptom in musculoskeletal disorders which likely explains these clear associations. The results also indicate that employees with chronic pain and obesity are at a particularly high risk of LTSA related to musculoskeletal disorders.

Social- and healthcare and education are, seen to the number of employees, the largest divisions within the City of Helsinki, and primary nurse, childminder and daycare teacher are among the most common work titles [38]. To further examine the role of employment sector to differences in pain, BMI and LTSA, additional analyses were performed separately for employees working in the social- and healthcare sector, education sector, and other sectors. Employees in the social- and healthcare sector had more LTSA than other sectors (Supplementary Table 3). However, this group did not have significantly more pain and overweight/obesity than the study population on average. We analysed associations between pain and sickness absence at different levels of BMI separately for the different employment sectors and found similar associations across the sectors, and similar to the results for the entire study population, suggesting that pain being more strongly associated with LTSA in employees with elevated BMI is a pattern observed across employment sectors.

Adjusting the analyses for sociodemographic factors, lifestyle factors and workload only marginally affected the results, indicating that they play a minor role to LTSA once pain and overweight/obesity are established. There should thus be focus on efforts to prevent pain and overweight/obesity in the younger working population, as it is known that pain in early life often persists into adulthood, similarly as overweight and obesity [39–41].

### Methodological considerations

The study is based on a large cohort of employees working in the public sector at the time of study entry, with a broad range of occupations [17]. Weight and height and the covariates were self-reported, which confers a risk of reporting bias. Participants’ overweight and obesity statuses were measured by BMI, which does not consider body composition and is consequently a controversial tool to measure adiposity. However, BMI is shown to correlate well with excess adiposity and self-reported BMI in population-level studies and is thus a valid indicator for assessing weight-related risks to work ability [42, 43]. Pain, instead, is a subjective phenomenon, and can reliably be measured in a survey.

The LTSA data were obtained from a national register comprising data over all sickness allowance periods from five years after the questionnaire was returned. The data collection was independent of the employment at the time of the follow-up, so change of employer within Finland did not interfere with the data collection. A limitation is that we were not able to account for that the actual follow-up for some of the employees may have been shorter due to, for instance, migration abroad or death; however, death is extremely rare in this age group [44]. Covariates were restricted to those enquired in the shorter phone interview, why information on for example physical activity was unavailable. Also, the study population consisted mainly of fertile-age women, why some respondents weight at baseline may have been affected by pregnancy. The data only comprised public sector employees and cannot be generalized to private sector employees [17]. Neither can the results be directly generalized to older employees, although similar associations between pain and obesity with sickness absence have been observed among older Finnish employees [12, 20]. The sample of respondents were found to be relatively representative of the target population, with a slightly higher proportion of highly educated employees [17]. The study was conducted among employees living in the capital region in Finland, where morbidity is lower and LTSA less common than the national average [45, 46].

## Conclusions

Young and early midlife employees with coexistent pain and overweight/obesity have an increased risk of LTSA, particularly employees with chronic pain and obesity. This finding highlights the importance of early prevention of pain and overweight/obesity and indicates that employees with coexistent pain and overweight/obesity could benefit from targeted primary and secondary preventive measures and the workplaces and in the occupational healthcare to reduce LTSA. 

## Supplementary Information

Supplementary Material 1.

Supplementary Material 2.

Supplementary Material 3.

## Data Availability

The Helsinki Health Study survey data cannot be made publicly available due to data protection laws and regulations. The data can only be used for scientific research. More information on the survey data can be requested from the Helsinki Health Study research group (kttl-hhs@helsinki.fi). The Helsinki Health Study study was launched in collaboration with the Whitehall II study and our surveys included largely the same measures. The Whitehall II questionnaires are published online at [https://www.ucl.ac.uk/brain-sciences/psychiatry/our-research/mental-health-older-people/whitehall-ii/data-collection]. Examples of survey items partly overlapping with the ones used here have also been published previously by Lallukka et al. 2025 [10.1371/journal.pone.0317010].
